# Cannabis sativa phytochemicals in cancer therapy: molecular mechanisms and therapeutic potential

**DOI:** 10.3389/fphar.2026.1768210

**Published:** 2026-06-23

**Authors:** Rosemary Montle, Garland K. More, Samkeliso Takaidza, Fanyana Mtunzi

**Affiliations:** 1 Institute of Chemical and Biotechnology, Vaal University of Technology, Sebokeng, South Africa; 2 College of Agriculture and Environmental Sciences, University of South Africa, Johannesburg, South Africa; 3 Department of Biotechnology, Vaal University of Technology, Vanderbijlpark, South Africa

**Keywords:** cancer therapy, cannabinoids, Cannabis sativa, endocannabinoid system, flavonoids, terpenes

## Abstract

**Background:**

The therapeutic potential of *Cannabis sativa* has attracted growing interest in oncology. Its diverse phytochemicals, including cannabinoids, flavonoids, and terpenes, interact with oncogenic signaling pathways and the endocannabinoid system influencing tumour progression and therapeutic responses.

**Objective:**

This review critically evaluates the molecular mechanisms by which *Cannabis sativa* phytochemicals modulate cancer pathways, with emphasis on apoptosis, oxidative stress regulation, autophagy, angiogenesis, and metastasis. It also explores synergistic and additive interactions among cannabinoids and flavonoids, highlighting their translational relevance.

**Key Findings:**

Cannabinoids such as Δ9-tetrahydrocannabinol (THC), cannabidiol (CBD), and cannabigerol (CBG) exhibit pathway-specific effects, including induction of apoptosis, modulation of oxidative stress, and inhibition of angiogenesis. Flavonoids such as cannflavin A, genistein, daidzein, hesperetin, and naringenin exhibit selective cytotoxicity across bladder, breast, melanoma, and pancreatic cancers, often sparing normal tissue. Importantly, phytochemical interactions are not uniformly synergistic; while combinations such as THC and CBD amplify apoptotic signaling, others act additively or antagonistically. Clinical formulations such as Nabiximols provide translational evidence of cannabinoid synergy, although outcomes remain context-dependent.

**Conclusion:**

The disconnect between preclinical efficacy and clinical outcomes underscores critical gaps in dosing strategies, patient selection, and combination regimens. Future research should prioritize mechanistic studies, rational phytochemical combinations, and innovative drug delivery systems. Taken together, *Cannabis sativa* phytochemicals emerge as promising molecular entities with the potential to reshape integrative oncology, provided their therapeutic promise is matched with rigorous, evidence-based evaluation.

## 1 Introduction

### 1.1 Cancer

Cancer encompasses a diverse category of diseases characterized by the uncontrolled proliferation and spread of atypical cells within the organism. These abnormal cells can develop into tumours, infiltrate adjacent tissues, and metastasize to distant regions through the lymphatic system. When left unchecked, this progression disrupts vital physiological functions and threatens the survival of humans (Anand et al., 2023). Globally, cancer is among the leading causes of death, accounting for nearly 10 million fatalities in 2020 (World Health Organization, 2020). It is one of two dominant non-communicable diseases responsible for millions of deaths annually (Li and Kuang, 2024). While cancers exhibit distinct genetic profiles, they share common molecular mechanisms and metabolic behaviours that enable their survival, growth and spread. The aetiology of cancer is complex and multifactorial, encompassing genetic mutations, environmental influences, lifestyle factors, and infections. Key contributors include exposure to carcinogens such as tobacco smoke and ultraviolet radiation, as well as inherited genetic predispositions and epigenetic changes that drive abnormal cell behaviour (Hassanpour and Dehghani, 2017). Chronic inflammation and viral infections, such as human papillomavirus (HPV) and hepatitis B virus (HBV), are also implicated in tumourigenesis (Mahmood and Srivastava, 2022). Research on tissue microenvironment and inflammation has advanced the understanding of tumour progression and treatment challenges. However, the underlying causes remain poorly understood, complicating efforts to achieve precise diagnosis and effective treatment (Upadhyay, 2021). The global burden of cancer continues to rise. In men, prostate, lung, and bronchus, colon and rectum, and urinary bladder cancers are most prevalent. For women, breast, colon, rectum, uterine corpus, lung and bronchus, and thyroid cancers dominate (GBD, 2023 Cancer Collaborators, 2025). Among children, blood cancers, as well as those affecting the brain and lymph nodes, account for the highest proportions. Notably, prostate cancer in men and breast cancer in women contribute significantly to the global cancer burden (Hassanpour and Dehghani, 2017).

The global burden of cancer continues to rise. In men, prostate, lung and bronchus, colon and rectum, and urinary bladder cancers are most prevalent. For women, breast, colon, rectum, uterine corpus, lung and bronchus, and thyroid cancers dominate. Among children, blood cancers, as well as those affecting the brain and lymph nodes, account for the highest proportions (World Health Organization, 2020; Hassanpour and Dehghani, 2017; Li and Kuang, 2024).

Despite advances in chemotherapy, targeted therapy, and radiation, treatment outcomes remain challenged by drug resistance, severe side effects, and limited long-term efficacy. These limitations have prompted growing interest in alternative approaches, including natural products such as cannabinoids and other phytochemicals from Cannabis sativa, which may offer complementary strategies in cancer therapy.

### 1.2 Phytochemicals in cannabis sativa

*Cannabis sativa L*., a dioecious member of the Cannabaceae family, produces a remarkably diverse array of phytochemicals. More than 500 compounds have been identified, including over 150 cannabinoids, ∼200 terpenes, and ∼20 flavonoids, alongside alkaloids and other minor metabolites (Tomko et al., 2020; LaVigne et al., 2021; Laezza et al., 2020). These molecules are defined not only by their chemical scaffolds but also by chromophore structural motifs, such as resorcinol cores, benzopyran rings, and conjugated double bonds, that mediate interactions with host proteins and receptors. Collectively, these phytochemicals underlie a broad spectrum of bioactivities, including antimicrobial, antiviral, antioxidant, anti-inflammatory, psychotropic, and anticancer effects.

**FIGURE 1:**
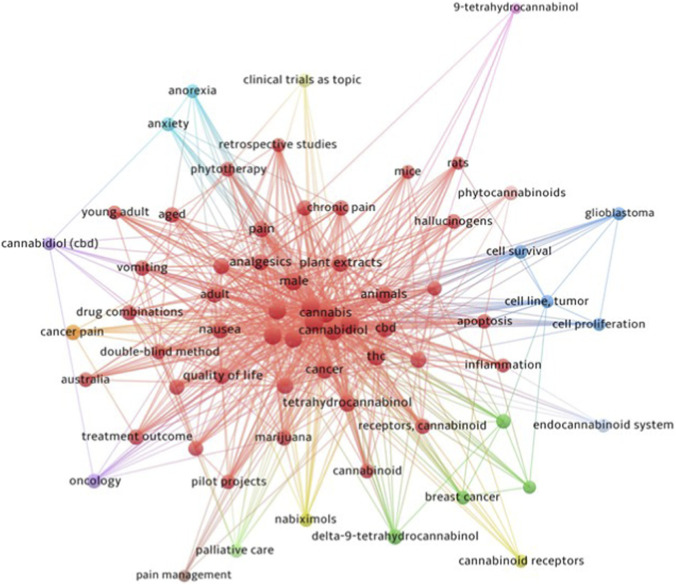
Network visualization of research trends and thematic clusters related to *Cannabis sativa* phytochemicals from 2002 to 2025, generated using VOSviewer software. Node size reflects keyword frequency, while colours indicate distinct thematic clusters.

Over the past 2 decades, the scientific conversation around *Cannabis sativa* has grown from niche pharmacology into a broad, multidisciplinary field. Researchers have explored its phytochemicals not only for their psychotropic effects but also for their roles in cancer biology, pain management, inflammation, and beyond. To capture this evolution, we mapped the literature using VOSviewer software. The visualization offers a bird’s-eye view of how research themes have clustered and shifted between 2002 and 2025. Larger nodes highlight the most frequently studied concepts, such as cannabinoids, cannabidiol, tetrahydrocannabinol, cancer, and pain, while coloured clusters reveal distinct thematic areas ranging from oncology to palliative care. Figure 1 provides a snapshot of the research landscape, showing that *Cannabis sativa* has become a focal point across diverse biomedical domains.

**FIGURE 2:**
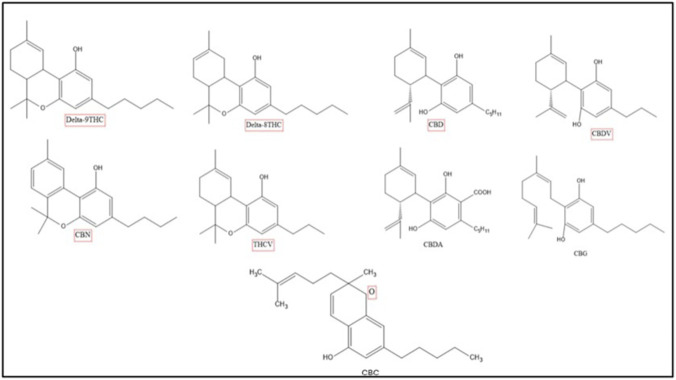
Chemical structures of selected cannabinoids from *Cannabis sativa L*., including Δ9-THC, Δ8-THC, CBD, CBDV, CBN, THCV, CBDA, CBG, and CBC. Structural diversity underpins their distinct biological effects in cancer and other pathologies.

To complement this bibliometric overview, representative chemical structures of the major cannabinoids are shown in Figure 2. These highlight the shared resorcinol and benzopyran motifs, alongside distinct substitutions and functional groups that contribute to their unique pharmacological activities.

#### 1.2.1 Phytochemicals

Across its diverse phytochemical repertoire, *Cannabis sativa* exhibits a broad bioactivity spectrum that extends beyond its well-known psychotropic effects.

**FIGURE 3:**
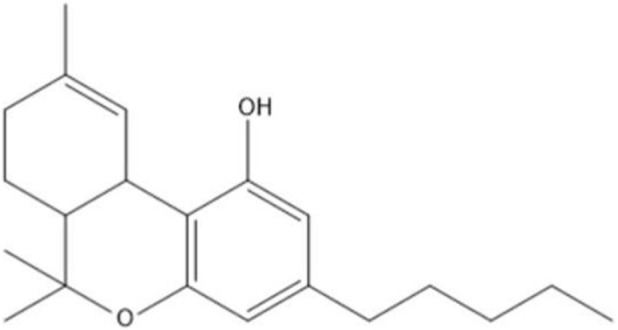
Chemical structure of Δ9-tetrahydrocannabinol (Δ9-THC), the principal psychoactive cannabinoid in *Cannabis sativa*. Drawn using ChemDraw for standardized bond thickness and atom spacing.

**FIGURE 4:**
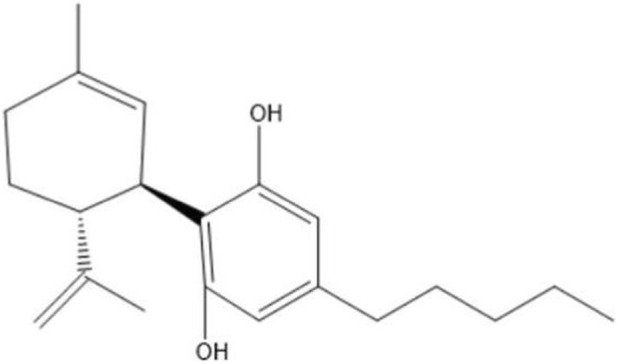
Chemical structure of cannabidiol (CBD), a non-psychoactive cannabinoid with a resorcinol core and hydroxyl substituents. Drawn using ChemDraw for clarity and consistency.

**FIGURE 5:**
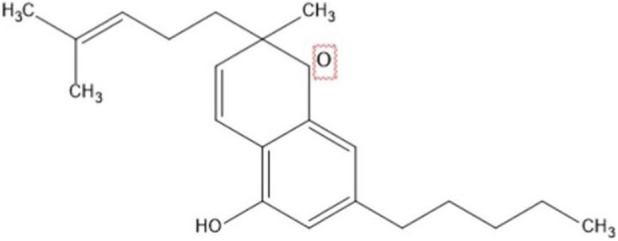
Chemical structure of cannabichromene (CBC), a non-psychoactive cannabinoid with anti-inflammatory and anti-proliferative properties. Drawn using ChemDraw to ensure standardized bond angles and atom spacing.

**FIGURE 6:**
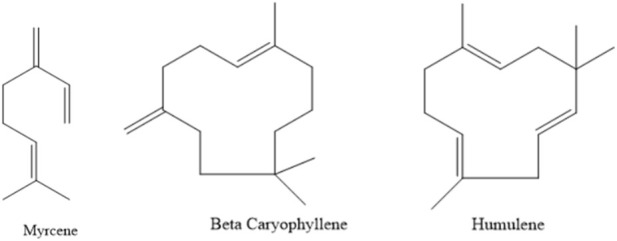
Chemical structures of three major terpenes in Cannabis sativa: myrcene, β-caryophyllene, and α-humulene. Myrcene is a monoterpene with sedative properties; β-caryophyllene is a CB2 agonist; α-humulene exhibits anticancer effects. Drawn using ChemDraw for structural clarity.

Cannabinoids such as Δ9-tetrahydrocannabinol (THC), cannabidiol (CBD), and cannabichromene (CBC) interact with host proteins including CB1, CB2, TRPV1, PPARγ, and NF-κB, with their resorcinol cores and benzopyran chromophores mediating these interactions (Janero et al., 2009; Ligresti et al., 2006; Calapai et al., 2022). Their chemical structures are shown in Figures 3–6, highlighting the shared motifs alongside distinct substitutions that confer unique pharmacological activities.

**FIGURE 7:**
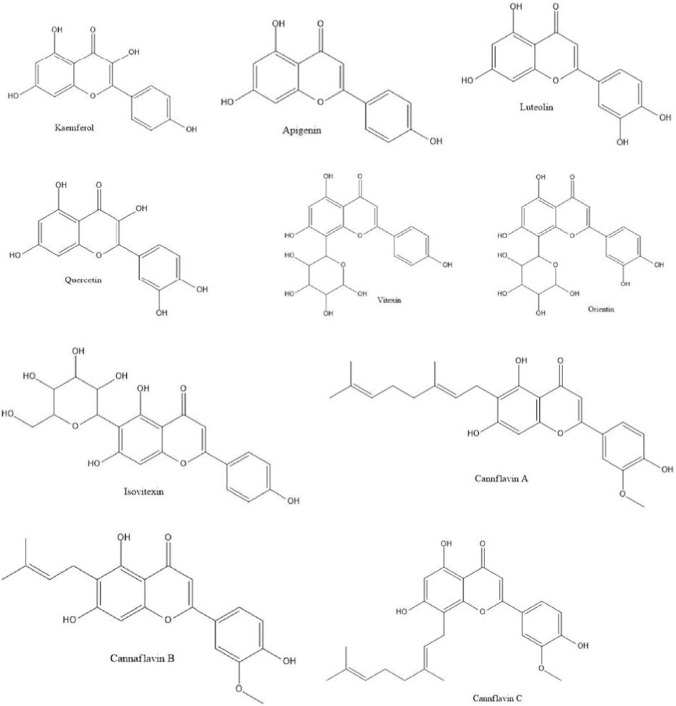
Chemical structures of flavonoids found in *Cannabis sativa*, including naringenin, apigenin, and cannflavins A and B. These compounds share a flavone backbone with substitutions influencing antioxidant and anticancer activity. Drawn using ChemDraw for standardized representation.

Terpenes, including β-caryophyllene and α-humulene, contribute to anti-inflammatory effects by downregulating NF-κB signaling and cytokines such as TNF-α and IL-6 (Tomko et al., 2020). Their structures, together with myrcene, are shown in Figure 7, illustrating the diversity of ring systems and functional groups that underpin their biological activity.

Flavonoids such as quercetin and naringenin, with their conjugated aromatic chromophores, fine-tune oxidative stress responses and modulate MAPK pathways, thereby supporting antiviral and antioxidant activity (Madureira et al., 2023). Cannabis-specific flavonoids, cannflavin A and B, exhibit potent anti-inflammatory effects. Their structures are shown in Figure 7, emphasizing the flavone backbone and variable hydroxyl and methoxy substitutions that influence antioxidant and anticancer properties.

Together, these phytochemicals illustrate how structural chromophores mediate diverse biological activities, ranging from antimicrobial and antiviral defence to psychotropic and anticancer modulation. This multifaceted spectrum underscores the pharmacophoric potential of *Cannabis sativa* and sets the stage for deeper exploration of its role in cancer signaling pathways. Table 1 summarizes selected phytochemicals, their reported anticancer activities, mechanisms of action, receptor interactions, and current clinical status, highlighting their translational relevance.

**TABLE 1:** Anticancer activities of selected *Cannabis sativa* phytochemicals.

Compound	Cancer type(s)	Mechanism of action	Efficacy data	Receptor activity	Clinical status	References
Cannabigerol (*CBG*)	Colon cancer, PDAC	*TRPM8* antagonism, ROS overproduction, ERK phosphorylation, *AKT/MTOR* inhibition, autophagy induction	Binding affinity: 10.2 kcal/mol; strong pro-apoptotic activity	Weak CB1/CB2 agonist, *TRPM8* antagonist	Preclinical	Kadriya et al. (2024); Zeppa et al. (2024)
Δ9-tetrahydrocannabinol (*THC*)	Glioblastoma, breast, lung cancer	CB1/CB2 activation, apoptosis induction, angiogenesis inhibition, *EGFR* downregulation	Ki: 40.7 nM (CB1), 36.4 nM (CB2); context-dependent effects	Partial CB1 agonist, full CB2 agonist	Clinical trials (palliative care)	Faiz et al. (2024); Koltai and Shalev (2022)
Cannabidiol (*CBD*)	Breast (MDA-MB-231), lung, neuroblastoma	CB2/*TRPV1* activation, Ca^2+^ elevation, ROS accumulation, *PI3K/AKT* modulation, *COX2/PPARG* activation	Docking scores: 7.9 (*TRPV1*), −7.8 (*TRPV4*), −7.5 kcal/mol (*TRPV2*); reduces invasion & metastasis	Low CB1/CB2 affinity, *TRPV1*, *PPARG*	Clinical trials ongoing	Li et al. (2023); Melo et al. (2025)
Cannabichromene (*CBC*)	Bladder, prostate, colorectal, breast cancer	Caspase-3/7 activation, Ca^2+^-dependent apoptosis, oxidative stress induction	IC_50_: 10–20 μM; synergy score: 38–71 with THC/CBD/CBV	Non-CB receptor pathways	Preclinical	Whynot et al. (2023); Tomko et al. (2020)
β-Caryophyllene (*BCP*)	Lung, ovarian, breast, osteosarcoma	CB2 agonism, *MAPK/PI3K/AKT/MTOR* modulation, *JAK1/STAT3* activation, ROS production	IC_50_: 137–270 μM (lung), 311–369 μM (breast), ∼20 μM (osteosarcoma)	Full CB2 agonist, *PPARG*	Preclinical	Di Sotto et al. (2020); Johnson et al. (2020)
Caryophyllene oxide (*CAO*)	Lung, colon, breast cancer	Mitochondrial membrane disruption, DOX synergy, apoptosis enhancement	IC_50_: 41 μM (lung), 235–298 μM (breast); enhanced DOX efficacy	CB2 pathway	Preclinical	Ambrož et al. (2019); Di Sotto et al. (2020)
α-Humulene (*HML*)	Hepatocellular carcinoma, prostate, pancreatic cancer	*AKT* inhibition, *GSK3/BAD* dephosphorylation, *NF-κB* downregulation, *BAX* upregulation	IC_50_: 11–17 μg/mL (HCC), 11 μg/mL (prostate); 75% inhibition with BCP	Non-cannabinoid pathways	Preclinical (*in vivo* validated)	Chen et al. (2019); Kang et al. (2022)
Cannflavin A	Bladder cancer	Cytotoxic activity, anti-inflammatory (30× aspirin potency)	IC_50_: 8 μM (T24), 15 μM (TCCSUP); 49%–80% cell death	Non-cannabinoid pathways	Preclinical	Tomko et al. (2022)
Quercetin	Breast cancer, various	ROS production, S/G2/M arrest, *BAX* upregulation, *BCL2* downregulation, *MMP2/9* inhibition	Disrupts cell cycle, limits metastasis	Non-cannabinoid pathways	Preclinical/clinical studies	Tubtimsri et al. (2025)
Naringenin	Melanoma (B16F10, SK-MEL-28)	ERK1/2 and JNK suppression, apoptosis and migration inhibition	100–400 μM: viability reduced to 43.8% (B16F10), 60.9% (SK-MEL-28)	Non-cannabinoid pathways	Preclinical	Choi et al., 2020
Hesperetin	Breast, colon cancer	G0/G1 and G2/M arrest, *HER2* downregulation, JNK pathway activation, ROS induction	10–500 μM: Modulates *TP53, NOTCH1*, β-catenin; inhibits *MMP9/RAC1*	Non-cannabinoid pathways	Preclinical	Sohel et al. (2022)

#### 1.2.2 Flavonoid-based anticancer activity across tumour types

Flavonoids, a diverse class of plant-derived compounds, are increasingly recognized for their selective anticancer properties. What makes them particularly compelling is their ability to target malignant cells while sparing healthy tissue, a feature that sets them apart from many conventional chemotherapeutics. Across different tumour types, flavonoids have shown consistent promise in modulating cell viability, apoptosis, oxidative stress, and metastatic signaling.

In bladder cancer, flavonoids such as cannflavin A, silymarin, luteolin, apigenin, and quercetin have demonstrated striking cytotoxicity against T24 cells, inducing 49%–80% cell death. Tomko et al. (2022) reported that cannflavin A achieved IC_50_ values of 8 µM in T24 cells and 15 µM in TCCSUP cells after 48 h, confirming its potency. Importantly, cannflavin A showed minimal toxicity in non-tumourigenic bladder epithelial cells, when compared to gemcitabine and cisplatin, which significantly reduced viability. This selectivity underscores the therapeutic potential of flavonoids as agents that can discriminate between healthy and malignant tissue.

This selectivity underscores the therapeutic potential of flavonoids as agents that can discriminate between healthy and malignant tissue. To contextualize these findings within the broader phytochemical spectrum, Table 2 compares the IC_50_ values of β-caryophyllene and caryophyllene oxide across different cancer models, highlighting how potency and synergy vary across phytochemical classes.

**TABLE 2:** Comparative IC50 Analysis of β-Caryophyllene (BCP) and Caryophyllene Oxide (CAO) across cancer models.

Cancer type	Cell line	BCP IC_50_ (µM)	CAO IC_50_ (µM)	Duration	References
Lung cancer	A549	137–270	41	48 h	Di Sotto et al. (2020)
Breast cancer	–	311–369	235–298	24 h	Di Sotto et al. (2020)
Osteosarcoma	MG-63	∼20	N/A	24 h	Di Sotto et al. (2020)
Lymphoblast leukemia	CCRF/CEM	311–369	235–298	24 h	Di Sotto et al. (2020)
Colon cancer	Caco-2	Synergistic with DOX*	Synergistic with DOX*	—	Ambrož et al. (2019)

Breast cancer studies further highlight the versatility of flavonoids. Kaushik et al. (2019) showed that genistein and Centchroman acted synergistically to induce G2/M arrest and inhibit PI3K/Akt signaling, thereby promoting ROS-dependent mitochondrial apoptosis. Kopustinskiene et al. (2020) described daidzein-mediated apoptosis in MCF-7 cells, marked by cytochrome c release, Bax/Bcl-2 modulation, and caspase activation. Jin et al. (2010) confirmed daidzein’s growth-suppressive effects, with IC_50_ values slightly above 100 µM after 72 h. Hesperetin has also emerged as a potent candidate: Sohel et al. (2022) demonstrated its ability to induce cell cycle arrest, apoptosis, and HER2 downregulation. At concentrations of 10–500 μM, hesperetin influenced p53, NOTCH1, β-catenin, and PPARG, while inhibiting MMP-9 and Rac1, impairing migration and viability. Interestingly, in colon cancer models, hesperetin activated JNK signaling, reduced COX-2 and CEA expression, and enhanced ROS-induced apoptosis, highlighting its cross-tumour relevance.

In melanoma, naringenin has shown consistent antitumour activity. Choi et al. (2020) reported that naringenin (100–400 µM) suppressed viability and migration in both B16F10 murine and SK-MEL-28 human melanoma cells, while inducing apoptosis. Viability in B16F10 cells dropped from 90.5% to 43.8%, and in SK-MEL-28 cells from 78.5% to 60.9%. Mechanistically, naringenin inhibited ERK1/2 and JNK signaling, particularly in B16F10 cells, where phosphorylated ERK1/2 and JNK were downregulated. These findings suggest that naringenin interferes with key survival pathways, positioning it as a promising candidate for melanoma therapy.

Pancreatic cancer research has also highlighted flavonoid innovation. Moreau et al. (2019) investigated Caflanone (FBL-03G), demonstrating its synergistic potential with radiotherapy. *In vitro*, Caflanone (1 µM) enhanced cell death in KPC and Panc-02 cell lines when combined with 4 Gy irradiation, while higher doses (4 µM) independently suppressed tumour growth. *In vivo*, SRB-mediated drug delivery improved tumour regression and survival, with abscopal effects extending therapeutic benefit systemically. This integration of flavonoid therapy with advanced delivery systems illustrates how natural compounds can be harnessed in modern oncology.

Taken together, these findings highlight flavonoids as versatile modulators of cancer biology. Their ability to induce apoptosis, regulate oxidative stress, and impair metastatic signaling, while sparing normal tissue, positions them as promising candidates for drug development. Future research should focus on dose optimization, rational combinations, and innovative delivery mechanisms to fully realize their therapeutic potential.

Having outlined the structural diversity and tumour-specific activity of cannabinoids, terpenes, and flavonoids, the following section explains the review methodology used to evaluate these findings.

### 1.3 Review method

#### 1.3.1 Oncogenic pathways

Cancer development is orchestrated by genetic mutations and dysregulated signaling cascades that drive uncontrolled proliferation, metastasis, and therapy resistance. A clear understanding of these pathways provides the foundation for targeted therapeutic strategies (Sarkar et al., 2013).

#### 1.3.2 Apoptotic pathways

Apoptosis is a central mechanism in tumour suppression, yet cancer cells frequently evade programmed cell death. Mutations in TP53, BCL2, and MYC disrupt intrinsic and extrinsic apoptotic signaling, enabling tumour survival. The mitochondrial pathway, regulated by BCL2 family proteins, includes pro-apoptotic members such as BAX and BAK that promote cytochrome c release and caspase activation, whereas anti-apoptotic proteins like BCL2 and BCL-XL counteract this process (Fulda and Debatin, 2006; Pistritto et al., 2016). The extrinsic pathway, mediated by death receptors such as FAS, TRAIL, and TNF, initiates caspase cascades but is often blocked by FLIP overexpression or receptor mutations. Oncogenic signaling via PI3K/AKT, RAS/RAF/MEK/ERK, and JAK/STAT further suppresses apoptosis, reinforcing tumour resilience.

#### 1.3.3 Oxidative stress and cancer regulation

Oxidative stress, defined as an imbalance between reactive oxygen species (ROS) and antioxidant defences, plays a paradoxical role in cancer. Chronic ROS accumulation promotes DNA damage, lipid peroxidation, and protein dysfunction, driving oncogenesis (Azmanova and Pitto-Barry, 2022; Forcados et al., 2017). Oncogenes such as RAS, RAC1, STAT3, BCL2, and MYC enhance ROS production through mitochondrial reprogramming and NADPH oxidase activation (Hayes et al., 2020). Loss of tumour suppressors such as TP53 reduces antioxidant gene expression, worsening oxidative stress. Tumour cells adapt by upregulating NADPH and glutathione synthesis, ensuring survival under oxidative pressure (Aggarwal et al., 2019). This duality of ROS as both a driver of malignancy and a therapeutic vulnerability underscores the importance of redox modulation in oncology (Muscolo et al., 2024).

#### 1.3.4 PI3K/AKT/mTOR pathway

The PI3K/AKT/mTOR axis is frequently hyperactivated in aggressive tumours, promoting proliferation, metabolic reprogramming, and chemoresistance. Dysregulation of this pathway enhances protein synthesis and inhibits apoptosis. Autophagy, regulated by AKT and mTOR, plays a dual role, functioning as both a tumour suppressor and promoter depending on context (Faiz et al., 2024). Downstream effects include CDKN1A (p21)-mediated cell cycle arrest, caspase activation, and altered metabolic signaling. Targeting PI3K/AKT/mTOR remains a cornerstone of precision oncology.

#### 1.3.5 Angiogenesis and metastasis

Angiogenesis and metastasis are hallmarks of cancer progression. Elevated ROS levels stimulate vascular endothelial growth factor (VEGFA) expression, driving tumour vascularization and epithelial-mesenchymal transition (EMT), which enhances motility and invasiveness (Kim and Byzova, 2014; Iqbal et al., 2024; Liu et al., 2025). Tumour cells adapt to oxidative stress by upregulating glutathione synthesis and other antioxidant defences, enabling survival in metastatic niches. Targeting ROS-mediated angiogenic signaling and EMT regulators offers therapeutic potential for limiting metastatic spread.

### 1.4 Cannabinoid modulation of oncogenic pathways

#### 1.4.1 Apoptotic pathways

Cannabidiol (CBD) promotes mitochondrial stress and caspase activation, while Δ9-tetrahydrocannabinol (THC) enhances mitochondrial permeability, together amplifying apoptotic signaling (Massi et al., 2013; Shrivastava et al., 2011). Cannabigerol (CBG) has been shown to suppress anti-apoptotic proteins, sensitizing tumour cells to programmed cell death (Borrelli et al., 2014).

#### 1.4.2 Oxidative stress regulation

CBD disrupts redox homeostasis by promoting ROS accumulation and impairing glutathione synthesis, selectively inducing apoptosis in cancer cells (Pagano et al., 2023; Pereira et al., 2021). Flavonoids such as naringenin fine-tune ROS levels to trigger apoptosis without harming normal cells (Madureira et al., 2023). THC and CBD also modulate transcription factors such as Nrf2, regulating antioxidant enzyme expression (Kopustinskiene et al., 2022).

#### 1.4.3 PI3K/AKT/mTOR pathway

THC and synthetic cannabinoids, including WIN-55,212–2, downregulate AKT signaling, inducing autophagy and apoptosis in glioma and lymphoma models (Faiz et al., 2024). Cannabinoid receptor activation promotes ceramide synthesis, which further suppresses AKT/mTOR signaling and enhances autophagic cell death (Moreno et al., 2014).

#### 1.4.4 Angiogenesis and metastasis

Cannabinoids inhibit VEGFA signaling, reducing angiogenesis and metastatic potential. CBD and THC downregulate hypoxia-inducible factor 1-alpha (HIF1A), limiting tumour vascularization (Tomko et al., 2020). Flavonoids modulate AMPK/PGC-1α signaling, disrupting cancer metabolism and reducing invasiveness (Ahmad et al., 2025).

### 1.5 Synergistic and additive interactions

The therapeutic potential of cannabinoids is not solely defined by their individual actions but by their interactions. Synergy occurs when combined effects exceed the sum of individual actions, whereas additive interactions yield outcomes equal to the sum. Importantly, not all cannabinoid interactions are synergistic; some are additive or even antagonistic (Russo, 2011).

THC and CBD demonstrate synergy in apoptosis regulation. CBD enhances ROS generation and mitochondrial dysfunction, while THC promotes mitochondrial permeability and caspase activation. Together, these mechanisms converge to amplify apoptotic signaling, particularly in glioblastoma models, where combined treatment reduced tumour viability more effectively than either compound alone (Massi et al., 2013; Shrivastava et al., 2011; Torres et al., 2011).

Additive interactions are evident in inflammatory pathways. Both CBD and CBG suppress NF-κB activation and cytokine release, but their combined administration does not exceed the expected sum of their effects (Borrelli et al., 2014). While not synergistic, additive outcomes remain clinically relevant, as they allow dose reduction of individual compounds, minimizing toxicity while maintaining efficacy.

At the receptor level, co-activation of CB1 and CB2 by THC, alongside CBD-mediated modulation of TRPV1 and GPR55 antagonism, produces convergent signaling that enhances anti-proliferative and anti-angiogenic responses (Moreno et al., 2014; Whyte et al., 2010). However, receptor cross-talk can also yield antagonistic outcomes, underscoring the complexity of cannabinoid pharmacodynamics.

Clinical formulations such as Nabiximols (a standardized THC: CBD extract) provide translational evidence of cannabinoid synergy. Initially approved for pain and spasticity, Nabiximols has demonstrated enhanced efficacy compared to isolated compounds, supporting the therapeutic relevance of synergistic cannabinoid interactions (Johnson et al., 2010). These findings emphasize the need for mechanistic studies to delineate when cannabinoid combinations yield synergistic, additive, or antagonistic outcomes.

Taken together, cannabinoid interactions are pathway-specific and context-dependent. Synergistic effects dominate in apoptosis and angiogenesis, while additive effects are more common in inflammation. Recognizing this distinction strengthens the argument for multi-component *cannabis*-based therapies in oncology, while cautioning against oversimplification of the “entourage effect” as universally synergistic.

## 2 Endocannabinoid system

**FIGURE 8:**
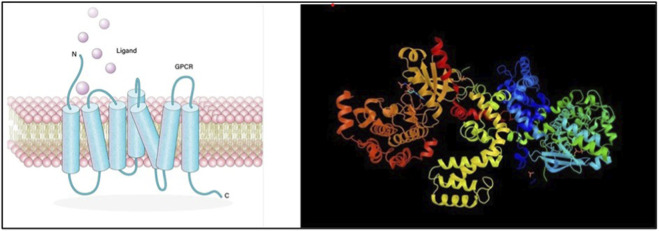
G-protein coupled receptors consisting of three domains, an extracellular domain containing the N-terminus where the ligand binds, the transmembrane helical domain (TMD) that crosses the membrane seven times, and the intracellular domain containing the C-terminus that activates the signalling proteins. (Image Credit: Udayadaithya M V/Shutterstock.com and ibreakstock/Shutterstock.com).

The endocannabinoid system (ECS) maintains homeostasis by regulating physiological processes such as appetite, pain sensation, mood, and inflammation across the endocrine, nervous, and immune systems. It consists of G-protein-coupled receptors (GPCRs), specifically CB1 and CB2, which possess a seven-transmembrane (7TM) domain structure and mediate the effects of cannabinoids through intracellular signalling pathways Figure 8. Additionally, the ECS includes endogenous ligands known as endocannabinoids, such as 2-arachidonoylglycerol (2-AG) and N-arachidonylethanolamide (AEA), which are lipid-derived molecules containing long-chain polyunsaturated fatty acids, esters, amides, and ethers. AEA partially activates CB1 and CB2 receptors, while 2-AG fully activates them (O’Brien, 2022). These ligands bind to CB1 and CB2 receptors, influencing neuronal activity, immune responses, and cellular metabolism. The system also relies on enzymatic regulation to maintain balance, with fatty acid amide hydrolase (FAAH) responsible for degrading AEA and monoacylglycerol lipase (MAGL) breaking down 2-AG to terminate its signaling effects. Research suggests that ECS dysregulation may contribute to various pathological conditions, including neurodegenerative diseases, inflammatory disorders, cancer progression, and metabolic syndromes (Chakravarti et al., 2014; Cherkasova et al., 2022).

**FIGURE 9:**
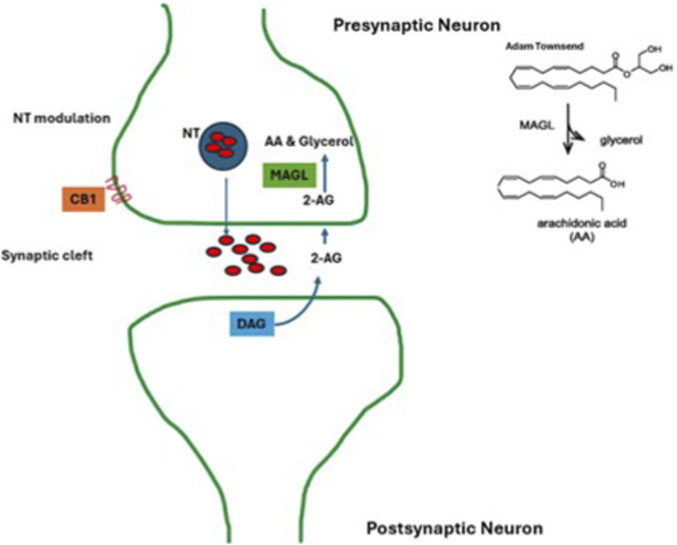
Enzymatic regulation of endocannabinoid signalling. Monoacylglycerol lipase (MAGL) hydrolyses 2-arachidonoylglycerol (2-AG) to terminate its signalling effects and maintain homeostatic balance. This degradation pathway ensures tight regulation of CB1 and CB2 receptor activation, preventing overstimulation of downstream signalling cascades.

CB1 receptors are highly expressed in the brain, particularly at the terminals of central and peripheral neurons, where they modulate neurotransmitter release and contribute to processes such as memory, cognition, analgesia, and motor function. They are also in peripheral organs and tissues where they regulate physiological processes, including energy metabolism, appetite control, and endocrine and metabolic functions. These roles make CB1 a promising therapeutic target for addressing various disorders (McAllister et al., 2021; Jung et al., 2018). In contrast, CB2 receptors are predominantly located in immune cells and peripheral tissues, including the spleen, tonsils, thymus, lungs, and macrophages, where they play a crucial role in regulating immune responses (McAllister et al., 2021). CB1 and CB2 receptors signal through fast and slow intracellular pathways, regulating neurological and immune functions. CB1, primarily in the central nervous system, controls fast ion currents like Ca^2+^ and K^+^, rapidly influencing neurotransmitter release, pain perception, and motor activity. CB2, mainly found in immune cells, engages slow signalling cascades, such as the cAMP-PKA pathway, modulating inflammation, immune responses, and cell survival. CB1 and CB2 receptors activate multiple intracellular signalling pathways, including PKA, PKC, MAPK, PI3K/Akt, JNK, ERK, and mTOR, depending on cell type and ligand interaction. These pathways regulate cell survival, apoptosis, gene expression, and metabolic functions. Cell growth or cell death results from specific receptor activation and the cellular environment (O’Brien, 2022). AEA and 2-AG are synthesized on demand in post-synaptic neurons from plasma membrane phospholipids, triggered by increased intracellular calcium or G-protein-coupled receptor activation. After being produced, they move backwards (retrogradely) to presynaptic CB1 receptors and inhibit neurotransmitter release, reducing neuronal excitation. This mechanism regulates synaptic activity, preventing overstimulation and maintaining neuromodulation (O’Brien, 2022). AEA and 2-AG then undergo degradation through their distinct enzymatic pathways Figure 9. Apart from CB1 and CB2, there are other phytocannabinoid receptors, namely, GPR55, TRPA, TRPV, and PPARs. These receptors interact with cannabinoids to regulate physiological processes (Koltai and Shalev, 2022).

The endocannabinoid system (ECS) has been implicated in cancer progression, with disruptions in metabolic regulation contributing to tumour growth and malignancy. Central metabolic regulators, including neuropeptide Y (NPY), which stimulates appetite, and CART peptide, which suppresses appetite, are involved in energy balance. Peripheral regulators comprising leptin (LEP), ghrelin (GHRL), adiponectin (ADIPOQ), and cholecystokinin (CCK) are known to be dysregulated in various cancers, influencing tumour metabolism and progression (Cherkasova et al., 2022). Studies have observed altered expression levels of CB1 and CB2 cannabinoid receptors, endogenous ligands such as anandamide (AEA) and 2-arachidonoylglycerol (2-AG), and variations in the activity of degrading enzymes, including fatty acid amide hydrolase (FAAH), monoacylglycerol lipase (MAGL), and N-acyl phosphatidylethanolamine phospholipase D (NAPE-PLD), in cancerous tissues (Gotfried et al., 2017). These molecular alterations suggest that ECS dysregulation may influence tumour growth, metastasis, and survival, making it a potential target for therapeutic intervention. Furthermore, Laezza et al. (2020) highlight that ECS dysfunction, characterized by changes in cannabinoid receptor expression, enzyme activity, and altered endocannabinoid levels, is associated with various pathological conditions, including cancer, neurodegenerative diseases, inflammatory disorders, multiple sclerosis, epilepsy, schizophrenia, cardiovascular diseases, glaucoma, and obesity. Such disruptions in ECS function compromise cellular homeostasis, underscoring its critical role in physiological regulation and its potential application in therapeutic strategies for disease management.

### 2.1 Changes in cannabinoid receptor expression profiles in cancer

Endocannabinoids, such as anandamide (AEA) and 2-arachidonoylglycerol (2-AG), interact with cannabinoid receptors CB1 and CB2 within the endocannabinoid system (ECS), regulating key physiological processes. Their activation can lead to upregulation or downregulation of CB1 and CB2 receptor expression in different cancer types, influencing tumour progression, apoptosis, and immune response (Cherkasova et al., 2022). However, studies investigating cannabinoid receptor regulation in cancerous tissues have yielded conflicting results, particularly regarding the CB2 receptor’s role in tumour progression (Ramer et al., 2021). Several reports highlight CB2 receptor upregulation in tumour tissues, including non-small cell lung cancer (NSCLC), squamous cell carcinoma of the head and neck, renal cell carcinoma, and HER2-positive breast cancer, linking this increase to cancer development and poorer survival outcomes. Additionally, studies indicate that HER2-CB2 receptor heteromers in breast tumours correlate with lower disease-free survival, reinforcing concerns that CB2 expression may contribute to malignancy. Similarly, reduced CB2 levels in tumour-associated macrophages from colorectal cancer patients have been associated with longer survival, suggesting a complex relationship between CB2 signaling and tumour immunity. Conversely, research in other cancer types presents a contrasting perspective. Higher CB2 receptor expression in lung cancer, hepatocellular carcinoma, and mobile tongue squamous cell carcinoma has been linked to improved survival, contradicting earlier findings that associated CB2 upregulation with poor prognosis (Preet et al., 2011; Ramer et al., 2021; Song et al., 2023). Pagano et al. (2021) noted discrepancies in the CB1 receptor expression in various cancers reported by different authors. While they reported a decrease, others found no alteration or increased CB1 receptor expression in high-grade glioma compared to low-grade glioma and healthy brain tissue. Another contradiction in human epithelial ovarian tumours, Messalli et al. (2014) reported that CB1 receptor expression progressively increases as tumours become more aggressive, transitioning from benign to borderline and ultimately to malignant, while Ronchi et al. (2021) reported that CB1 receptor expression varies among malignant epithelial ovarian tumours based on the dualistic model of ovarian carcinogenesis. In highly aggressive Type II ovarian tumours, CB1 receptor expression is predominantly negative or weak. The involvement of CB1 and CB2 receptors in cancer appears to be tumour-dependent, with their role varying across different malignancies. Their influence may promote or inhibit disease progression based on the cellular context. These observations highlight the necessity for further research into CB receptor modulation and its effects on tumour biology.

### 2.2 Metabolomics and cancer: Insights from phytochemical modulation

The application of metabolomics has significantly advanced our understanding of metabolic alterations in cancer, shedding light on the biochemical processes that sustain tumour growth, survival, and resistance to treatment. By examining biological samples such as tissues, cells, and biofluids (plasma, urine, serum, and saliva), researchers can characterize metabolic shifts that contribute to disease progression (Armitage and Barbas, 2014). Cancer cells undergo extensive metabolic reprogramming, modifying fundamental pathways, glycolysis, lipid metabolism, and oxidative stress regulation, to support unchecked proliferation and evade apoptosis (Tufail et al., 2024). These metabolic adaptations present potential therapeutic targets, particularly through the modulation of tumour metabolism by phytochemicals derived from *Cannabis sativa L*. Among these bioactive compounds, cannabinoids, flavonoids, and terpenes have demonstrated effects on tumour metabolism, influencing key regulatory processes. Δ^9^-Tetrahydrocannabinol (THC) has been identified as a modulator of glycolytic flux, which may restore metabolic equilibrium in malignancies (Chayasirisobhon, 2021). Beyond its role in energy metabolism, THC interacts with lipid signaling mechanisms, particularly ceramide biosynthesis, a pathway central to apoptotic regulation in glioma cells. By enhancing serine palmitoyltransferase (SPT) activity, THC facilitates ceramide accumulation, triggering apoptosis, an effect that can be reversed through inhibition of *de novo* ceramide synthesis. Furthermore, THC modulates extracellular signal-regulated kinase (ERK) activation and protein kinase B (Akt) suppression, which impairs survival signaling (Gomez del Pulgar et al., 2002). These findings highlight THC’s therapeutic potential in targeting lipid-dependent cancer pathways. Another class of phytochemicals, flavonoids such as quercetin, are known to mediate oxidative stress pathways, enhancing cancer cell susceptibility to apoptosis. Studies indicate that quercetin-induced ROS accumulation can disrupt cell cycle progression at the S and G2/M phases, concurrently limiting metastatic potential through its effects on cell migration and invasion. At the molecular level, quercetin promotes BAX-mediated apoptotic signaling while downregulating anti-apoptotic factors BCL-2/BCL-XL, reinforcing its tumour-suppressive effects. Additionally, quercetin influences extracellular matrix remodeling, increasing TIMP-1 levels and reducing MMP-2/MMP-9 activity, thereby hindering metastatic progression (Tubtimsri et al., 2025). In addition to cannabinoids and flavonoids, terpenes contribute to metabolic modulation in cancer therapy. Carvacrol, delivered as a nanoemulsion, has exhibited strong anticancer activity against human lung adenocarcinoma, primarily by amplifying mitochondrial ROS production, leading to apoptosis. Likewise, cacalol, another terpene, alters lipid metabolism in breast cancer by downregulating fatty acid synthase (FAS), thereby disrupting tumour lipid biosynthesis and inducing apoptosis via DAPK2 and caspase-3 activation (Pandey et al., 2025).

The implementation of metabolomics techniques, such as high-performance liquid chromatography-mass spectrometry (HPLC-MS) and gas chromatography-mass spectrometry (GC-MS), has enabled the identification of cannabis-derived metabolic signatures associated with cancer therapeutics (Pourseyed Lazarjani et al., 2020). These approaches provide valuable insights into ceramide-mediated apoptosis mechanisms induced by cannabinoids and flavonoid-mediated oxidative stress modulation, highlighting their impact on cancer cell vulnerability. Metabolomics is advancing cannabis-based oncology research, offering precision medicine opportunities by using phytochemical bioactivity to target key metabolic pathways in cancers.

## 3 Endocannabinoid system and *Cannabis sativa* phytochemicals

### 3.1 Historical and ethnopharmacological perspective

The foundational understanding of the endocannabinoid system (ECS) in human physiology has roots in empirical observations of *Cannabis sativa* consumption. Belonging to the Cannabaceae family, *Cannabis sativa* is one of the earliest domesticated crops, historically valued for its fibers, seeds, and medicinal properties. Its therapeutic application dates back approximately 5,000 years, when it was employed in traditional medicine to treat ailments such as fatigue, rheumatism, malaria, and eczema. By the 19th century, cannabis had entered modern Western medicine as an analgesic, anticonvulsant, anti-inflammatory, and antiemetic agent. However, restrictive regulations introduced in the 1930s in Western Europe and the United States curtailed its medical use, shaping the current global legal landscape (Capodice and Kaplan, 2021).

### 3.2 Structural and functional components of the ECS

The endocannabinoid system (ECS) comprises endogenous lipid-based ligands (N-arachidonoylethanolamide [AEA] and 2-arachidonoylglycerol [2-AG]), G-protein-coupled cannabinoid receptors (CB1 and CB2), and enzymes such as fatty acid amide hydrolase (FAAH) and monoacylglycerol lipase (MAGL) that mediate their biosynthesis and degradation. CB1 receptors are primarily distributed in the central nervous system and modulate memory, pain, and appetite, whereas CB2 receptors are found predominantly in immune and peripheral tissues, where they regulate inflammatory responses (Di Marzo and Piscitelli 2015). The endogenous ligands possess long hydrophobic tails and terminal amide or ester groups that enable interactions with the lipid-facing surfaces of CB1 and CB2. These interactions include hydrogen bonding within receptor transmembrane domains, contributing to the stabilization and activation of receptor conformation. Beyond CB1 and CB2, other receptors such as GPR55 and TRPV1 are implicated in cannabinoid signaling, broadening the physiological scope of ECS modulation (Almogi-Hazan and Or, 2020).

### 3.3 Pharmacophoric properties of *cannabis sativa* phytochemicals

*Cannabis sativa* produces over 500 bioactive compounds, including cannabinoids, terpenoids, and flavonoids, which interact with ECS components. Their biological activity can be traced to distinct pharmacophoric elements that determine receptor binding and efficacy.

Δ9-Tetrahydrocannabinol (THC), the primary psychoactive compound in *Cannabis sativa*, acts as a partial agonist at CB1 and a full agonist at CB2. Its phenolic hydroxyl group forms hydrogen bonds, while its pentyl side chain engages in hydrophobic interactions with CB1’s orthosteric binding site. Structural docking studies have shown that THC’s side chain penetrates a hydrophobic sub-pocket of CB1, enhancing binding stability and receptor activation (Jung et al., 2018).

Tetrahydrocannabivarin (THCV), a propyl analog of THC, exhibits concentration-dependent receptor behaviour: it antagonizes CB1 at low doses and partially activates CB2 at higher concentrations. Its shorter alkyl side chain reduces hydrophobic binding affinity within CB1, accounting for its antagonist properties and reduced psychoactivity (Shahbazi et al., 2020; Jung et al., 2018).

Although cannabidiol (CBD) has low affinity for CB1 and CB2 receptors, it modulates the ECS indirectly by inhibiting FAAH and influencing non-cannabinoid receptors such as TRPV1 and 5-HT1A. Its pharmacological profile includes anti-inflammatory, neuroprotective, and anxiolytic effects, making it a versatile therapeutic candidate (Almogi-Hazan and Or, 2020).

β-Caryophyllene (BCP) is a bicyclic sesquiterpene that selectively binds to CB2 receptors. Its hydrophobic scaffold allows stable binding within the CB2 transmembrane region, and its conjugated double bonds further enhance binding affinity. Importantly, BCP also acts as a PPARγ agonist, implicating it in metabolic regulation and anti-inflammatory pathways (Sharma et al., 2016; Baradaran Rahimi et al., 2023).

### 3.4 Physiological and therapeutic implications

The phytochemical and endocannabinoid system (ECS) interactions regulate inflammation, pain, mood, metabolism, and neurodegeneration. THC and CBD have demonstrated efficacy in managing chronic pain and epilepsy, while THCV and BCP are under investigation for metabolic syndrome and inflammatory diseases, respectively. These therapeutic actions are attributed to their precise molecular interactions within receptor binding domains and downstream signaling pathways (Di Marzo and Piscitelli, 2015; Almogi-Hazan and Or, 2020).

### 3.5 Synergistic effects and therapeutic optimization

The therapeutic efficacy of *Cannabis sativa* is enhanced by the “entourage effect,” a synergistic interaction among its phytochemicals. This phenomenon suggests that whole-plant extracts may be more effective than isolated compounds due to cumulative receptor modulation and secondary pathway activation (Almogi-Hazan and Or, 2020).

## 4 Mechanisms of action

Cannabinoids such as cannabidiol (CBD) and tetrahydrocannabinol (THC) exert significant biological effects through interactions with the endocannabinoid system (ECS), influencing reproductive function, bladder physiology, and cancer progression. These compounds modulate key molecular pathways by binding to cannabinoid receptors (CB1 and CB2), impacting cell cycle regulation, immune response, and angiogenesis. In the male reproductive system, ECS components are present in the testes, seminal vesicles, spermatozoa, and corpus cavernosum, regulating spermatogenesis and neurotransmitter release. However, excessive cannabis use may inhibit sperm function due to elevated anandamide (AEA) levels (Capodice and Kaplan, 2021). Cannabinoids also suppress tumour proliferation by interfering with cyclins and cyclin-dependent kinases (CDKs), inducing cell cycle arrest and inhibiting survival pathways such as PI3K/Akt and MAPK (Erhabor et al., 2024). Their immunomodulatory effects enhance immune surveillance by promoting natural killer (NK) cell and cytotoxic T lymphocyte activity while reducing pro-inflammatory cytokine production, creating an unfavourable environment for tumour progression. In the urinary bladder, CB1 and CB2 receptors are expressed in the urothelium and detrusor muscle, allowing cannabinoids to influence voiding dysfunction by reducing nerve growth factor (NGF) signaling and inhibiting adenylyl cyclase, resulting in analgesic effects and improved lower urinary tract symptoms (LUTS) in multiple sclerosis (MS) patients (Capodice and Kaplan, 2021). Furthermore, cannabinoids exert anti-angiogenic effects by suppressing vascular endothelial growth factor (VEGF) signaling, thereby reducing tumour vascularization and limiting cancer progression, with studies showing the Eastern Cape extract exhibits stronger VEGF inhibition than the Lesotho extract with high nitric oxide (NO) inhibitory effects, peaking at 91% (EC extract, 1.6 μg/mL), further limit angiogenic processes. The identification of cannabidiol (CBD) and tetrahydrocannabinolic acid (THCA) through high-performance liquid chromatography (HPLC) confirms their contribution to these mechanisms (Erhabor et al., 2024). Recent proteomic analyses indicate cannabis use alters protein expression profiles in urine biomarkers, affecting lipid metabolism, immune function, and tumour-related processes, demonstrating potential oncologic implications. While cannabis use presents reproductive and oncologic risks, its therapeutic potential in bladder dysfunction and cancer management warrants further research to refine bioavailability, optimize efficacy, and expand clinical applications.

## 5 Molecular docking vs. *cannabis* phytochemicals in cancer therapy

**FIGURE 10:**
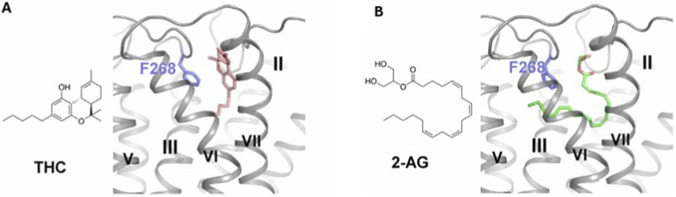
Chemical structures and predicted binding poses of Δ9-tetrahydrocannabinol (THC) (pink sticks, panel **(A)**) and 2-arachidonoylglycerol (2-AG) (green sticks, panel **(B)**) within the cannabinoid receptor binding pocket. THC is shown interacting with hydrophobic residues, while 2-AG demonstrates a distinct orientation consistent with its role as an endogenous ligand. Structural modelling adapted from Hua et al. (2016).

**FIGURE 11:**
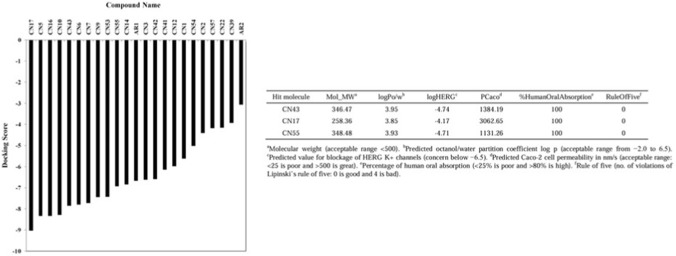
Clustered column chart showing docking scores of 21 cannabinoids (CNs) and standards (AR1 and AR2), alongside a table summarizing the ADMET profiles of the most promising compounds.

Research on *Cannabis sativa L*. has investigated various metabolite classes, including cannabinoids, flavonoids, terpenes, lignanamides, and alkaloids, through molecular docking studies. These bioactive compounds have been assessed for their interactions with enzymes that play a role in essential physiological processes, such as digestion, hormone regulation, and nerve signaling. Binding affinities, typically measured in kcal/mol, indicate the strength of ligand-receptor interactions, where lower values correspond to higher binding affinity (Hourfane et al., 2023). Representative docking poses of Δ9-THC and 2-AG within the CB1 receptor binding pocket are shown in Figure 10, highlighting their interactions with residue F268 and surrounding helices. Baroi et al. (2020) studied molecular docking of *Cannabis sativa* metabolites with aromatase, an enzyme involved in hormone-sensitive cancers like breast and ovarian cancer. They compared the binding potential of these metabolites to standard inhibitors such as fadrozole (AR1) and androstenedione (AR2) Figure 11. Their docking analysis showed that all tested compounds formed at least one hydrogen bond with Met374, a key active-site residue. Differences in inhibitory activity were influenced by steric hindrance and interactions with heme Fe^3+^. Hydrophobic interactions with amino acid residues and a heme group (Ala306, Trp224, Val369, Val370, Ile133, and Phe134) stabilized ligand binding, aligning with findings on non-steroidal aromatase inhibitors. Of the 61 cannabinoids screened, 21 exhibited docking scores comparable to standard inhibitors, while 14 surpassed a threshold of 6, indicating strong binding affinity. Compounds cannabidiorcol (CN 17), cannabitriol (CN 43), and cannabiripsol (CN 55), which interact directly with heme Fe^3+^, demonstrated enhanced inhibitory effects, reinforcing their potential in cancer treatment therapy. Drug-likeness evaluations using Lipinski’s Rule of Five identified cannabidiorcol (CN 17), cannabitriol (CN 43), and cannabiripsol (CN 55) as promising candidates. ADMET analysis confirmed their favourable pharmacokinetic properties, including molecular weight, logPo/w, logHERG, and human oral absorption, supporting their therapeutic potential. Mass spectrometry validated their identities with m/z values of 259.352, 347.485, and 349.365, respectively. These findings from Baroi et al. (2020) emphasize their strong binding interactions within the aromatase active site, particularly with the heme moiety, reinforcing their relevance in oncology research.

Molecular docking analysis by Melo et al. (2025) using CB-Dock2 examined the interactions between cannabidiol (CBD) and multiple receptors, including vanilloid receptors (TRPV1, TRPV2, and TRPV4), cannabinoid receptors (CB1 and CB2), and VDAC1. CBD demonstrated high binding affinity with vanilloid receptors, with docking scores of −7.9 kcal/mol for TRPV1, −7.8 kcal/mol for TRPV4, and −7.5 kcal/mol for TRPV2. Key amino acid interactions were identified across receptor chains, particularly PHE43, ASN438, PHE587, VAL441, and PHE591 for TRPV1, and TYR439, PHE471, ASN474 for TRPV4. Despite TRPV2 exhibiting the lowest docking score, it still showed strong affinity and has been linked to cancer cell death mechanisms. Additionally, studies suggest that CBD’s activity may be mediated through CB1, CB2, and VDAC1, broadening its therapeutic potential.

**FIGURE 12:**
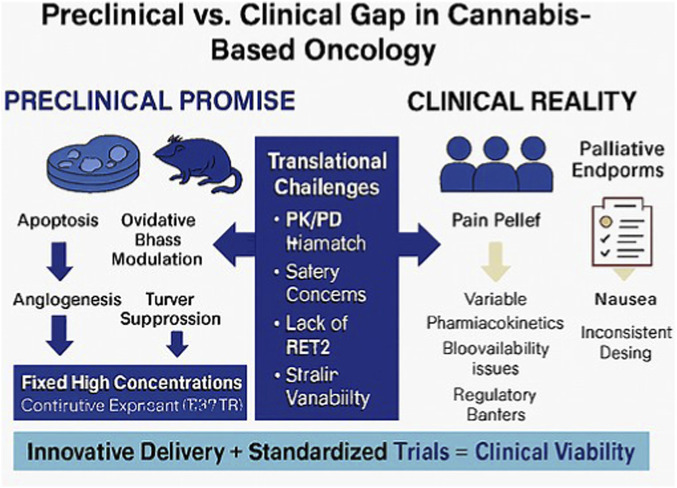
Binding modes of top-ranked lignanamides in the drug-binding pocket of P-glycoprotein (P-gp). Cannabisin N (blue sticks) and Cannabisin M (green sticks) occupy distinct orientations within the transmembrane domain. Cannabisin N demonstrates hydrogen bonding with key residues, while Cannabisin M exhibits hydrophobic interactions that stabilize its binding pose. These predicted docking conformations highlight potential inhibitory activity against P-gp-mediated drug efflux.

Kazemi et al. (2021) reported on the interactions between *Cannabis sativa* lignanamides and P-glycoprotein (P-gp), a key transporter in drug resistance, using molecular docking. Their study revealed that Cannabisin M and Cannabisin N exhibited higher binding affinities (−10.2 kcal/mol) within P-gp’s drug-binding pocket, outperforming the standard inhibitors tariquidar (−10.1 kcal/mol) and zosuquidar (−9.6 kcal/mol) Figure 12. Binding analysis showed that Cannabisin M formed hydrophobic interactions with Ile864, Tyr949, Phe339, Phe332, Phe728, Leu335, Leu64, Val978, and Phe974, alongside hydrogen bonds with Tyr306 and Ile336. Likewise, Cannabisin N engaged in hydrophobic interactions with Phe974, Ser975, Phe724, Gln721, Tye303, Tyr306, and Phe332, supported by a hydrogen bond with Tyr949, reinforcing its binding stability.

Further ADME profiling assessed their drug-likeness using Lipinski’s Rule of Five, which evaluates molecular weight, hydrogen bonding, and lipophilicity. While Tariquidar, Zosuquidar, Grossamide, Cannabisin-F, and Cannabisin-N adhered to most criteria, some compounds exceeded 500 Da molecular weight, leading to violations. Among them, Zosuquidar demonstrated high gastrointestinal (GI) absorption and the ability to cross the blood-brain barrier (BBB), distinguishing it from the rest.

## 6 Preclinical and clinical findings

*Cannabis sativa*-derived compounds have attracted considerable research interest in oncology, largely because of their ability to modulate multiple cancer pathways simultaneously. Preclinical studies show that cannabinoids, flavonoids, and terpenes can trigger apoptosis, modulate oxidative stress, inhibit tumour growth, and block angiogenesis (Kopustinskiene et al., 2020).

Cannabinoids, such as CBD and THC, engage CB1 and CB2 receptors, influencing oncogenic pathways like PI3K/AKT/mTOR and MAPK/ERK, ultimately reducing tumour viability and enhancing immune response (Tomko et al., 2020). Flavonoids, including quercetin, kaempferol, and cannflavins, exert antioxidant and anti-inflammatory effects, disrupting cancer cell metabolism and VEGF-mediated angiogenesis (Whynot et al., 2023). Terpenes, β-caryophyllene and α-humulene act on apoptotic mechanisms by triggering ROS accumulation, leading to cancer cell death (Johnson et al., 2020).

Despite promising preclinical evidence, clinical trials primarily focus on symptom management rather than direct tumour suppression, with cannabinoids showing efficacy in pain relief, nausea reduction, and appetite stimulation (Aly et al., 2019). Limitations in clinical integration include bioavailability issues, standardization challenges, and regulatory constraints, necessitating further research to optimize drug formulations through nanotechnology-enhanced delivery systems (Kopustinskiene et al., 2020).

Additionally, synergistic effects between cannabinoids and conventional therapies, such as chemotherapy or radiotherapy, require evaluation to determine potential benefits in overcoming drug resistance (Tomko et al., 2020). Moving forward, studies should investigate standardized dosing regimens, conduct large-scale randomized controlled trials, and refine target-specific applications of cannabis-based therapies. Incorporating C. sativa compounds into oncology could significantly advance personalized cancer treatment, offering novel therapeutic strategies that complement existing oncological interventions while minimizing toxicity and improving patient outcomes.

## 7 Challenges and limitations

The integration of *Cannabis sativa*-derived compounds into oncology presents both promising opportunities and notable challenges. Preclinical studies suggest that cannabinoids, flavonoids, and terpenes possess anticancer properties, exerting effects such as apoptosis induction, oxidative stress modulation, inhibition of tumour proliferation, and suppression of angiogenesis (Kopustinskiene et al., 2020). However, a lack of standardized dosing protocols remains a significant barrier to clinical application. Unlike conventional chemotherapeutic agents, cannabinoid-based therapies lack established pharmacokinetic and pharmacodynamic profiles, making it difficult to determine therapeutic concentrations across different cancer types (Tomko et al., 2020). Moreover, the high variability in *cannabis* composition due to differences in strain genetics, cultivation methods, and extraction techniques further complicates reproducibility in studies. The potency and therapeutic efficacy of C. sativa compounds vary depending on the ratio of cannabinoids, flavonoids, and terpenes, creating inconsistencies in research findings and patient responses (Whynot et al., 2023). Regulatory and ethical challenges also pose significant limitations to research and clinical implementation. In many countries, *cannabis* remains classified as a controlled substance, restricting funding, access, and large-scale clinical trials (Johnson et al., 2020). While some regions have moved toward legalization for medical use, there is still insufficient guidance for oncologists regarding safe and effective prescribing practices.

## 8 Safety concerns and adverse effects

Cannabis-derived compounds generally show good tolerability in clinical studies, but cancer patients present unique safety considerations. These patients are often immunocompromised and taking multiple medications simultaneously, creating potential for complications not seen in healthy preclinical models. THC’s psychoactive properties are particularly concerning in this population. Cognitive impairment, reduced coordination, and altered judgment pose serious risks for patients with brain metastases or those already experiencing neurotoxicity from chemotherapy. These effects could significantly impact quality of life and functional independence when layered on top of existing treatment-related symptoms. Drug interactions add another layer of complexity. Cannabinoids undergo metabolism primarily through cytochrome P450 enzymes, especially CYP3A4 and CYP2C19, creating potential interactions with chemotherapy agents, immunosuppressants, and anticoagulants commonly prescribed in oncology (Johnson et al., 2020). These interactions might reduce the efficacy of critical medications or increase their toxicity. Long-term safety data remain surprisingly limited. While acute toxicity studies suggest cannabinoids are relatively safe at therapeutic doses, chronic high-dose exposure effects on endocrine function and reproductive health are poorly understood. More concerning is the possibility that sustained cannabinoid use might suppress immune surveillance mechanisms in ways that could theoretically promote tumour growth (Capodice and Kaplan, 2021). The CB2 receptor expression data discussed earlier in this review reinforce these concerns. CB2 upregulation correlates with better outcomes in some cancers but worse prognosis in others, depending on tumour type (Ramer et al., 2021). This suggests that cannabinoid effects are highly context-dependent potentially beneficial in one cancer type but harmful in another. Without better mechanistic understanding, broad cannabinoid therapy application carries risks that current evidence cannot fully characterize.

Methodological challenges further impede progress, as current research lacks large-scale randomized controlled trials necessary to establish robust clinical evidence. Most studies focus on palliative care applications, such as pain relief, appetite stimulation, and nausea reduction, rather than direct anticancer effects (Tomko et al., 2020). Furthermore, the bioavailability of cannabinoids is a crucial limitation, as oral formulations often exhibit inconsistent absorption rates, affecting therapeutic outcomes (Kopustinskiene et al., 2020). To overcome these challenges, future research should focus on optimizing extraction techniques, developing targeted drug delivery systems such as nanoparticle formulations, and evaluating synergistic effects with conventional therapies (Whynot et al., 2023). Expanding clinical trials to assess the efficacy of cannabinoids in direct tumour suppression will be essential for establishing cannabis-based interventions as viable oncology treatments.

Additionally, creating standardized dosing guidelines and enhancing physician training will ensure safe and effective implementation. If these limitations are adequately addressed, *Cannabis sativa* could become a valuable adjunct therapy, complementing traditional oncology approaches while minimizing drug resistance and toxicity. In conclusion, while significant hurdles remain, the potential of cannabis-derived compounds in oncology justifies continued investigation and strategic advancements to harness their therapeutic benefits fully (Johnson et al., 2020).

## 9 Reconciling preclinical promise with clinical reality

The gap between promising preclinical results and the limited clinical use of *Cannabis sativa*-derived anticancer therapies needs careful evaluation. Several interconnected factors may explain this disconnect.

### 9.1 Pharmacokinetic-pharmacodynamic mismatch

One of the most significant barriers to clinical translation is the gap in concentration between preclinical efficacy and achievable systemic exposures. Throughout this review, we have documented effective *in vitro* concentrations that raise questions about clinical feasibility. Cannabichromene (CBC), for example, required 10–20 μM to induce cytotoxic effects across multiple cancer cell lines, with caspase 3/7 activation most pronounced at 20 μM in prostate cancer models (Tomko et al., 2020). β-Caryophyllene (BCP) showed IC_50_ values of 137–270 μM in lung cancer and 311.2–368.5 μM in breast cancer after 24-h exposure (di Sotto et al., 2020). Naringenin needed 100–400 μM for anticancer activity in melanoma cells (Choi et al., 2020). Even more potent compounds face challenges—cannflavin A (IC_50_ = 8 μM in T24 bladder cancer cells; Tomko et al., 2022) and caryophyllene oxide (IC_50_ = 41 μM in lung cancer; di Sotto et al., 2020) still require sustained tissue concentrations that may be difficult to achieve through conventional oral or intravenous administration.

Cannabinoid pharmacokinetics make the problem worse. These lipophilic compounds undergo extensive first-pass metabolism, and absorption varies considerably based on formulation, whether patients have eaten, and individual metabolic differences (Kopustinskiene et al., 2020). Most preclinical studies use 24–72-h continuous exposure at fixed concentrations under conditions that do not reflect real-world pharmacokinetics, where drug levels rise and fall. Achieving micromolar concentrations systemically could require doses that cause unacceptable psychoactive effects (for THC) or other toxicities, severely limiting therapeutic windows. Oral formulations show particularly inconsistent absorption, making it nearly impossible to maintain therapeutic tumour concentrations while avoiding systemic toxicity (Kopustinskiene et al., 2020; Tomko et al., 2020).

This gap between preclinical promise and clinical reality points to the critical need for better delivery systems. Nanotechnology-based formulations, liposomal encapsulation, and targeted delivery approaches could increase tumour accumulation while reducing systemic exposure. Local or regional delivery, intratumoral injection, or convection-enhanced delivery for brain tumours may bypass systemic limitations entirely and deserve more research attention.

**FIGURE 13:**
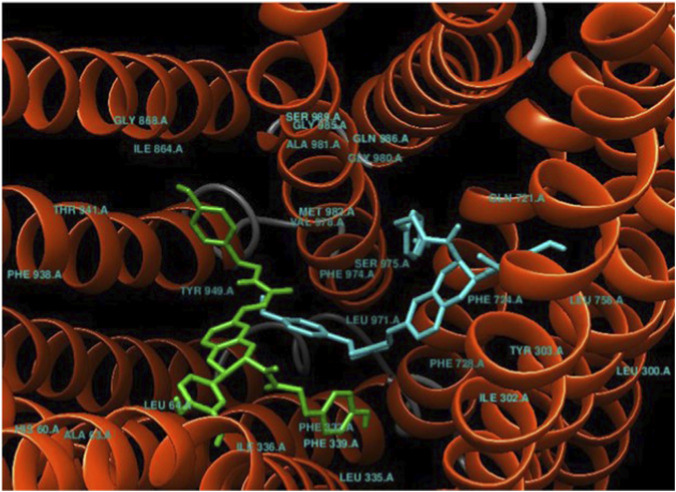
Schematic of the translational gap between preclinical promise and clinical reality in *Cannabis sativa* oncology research. Preclinical models show anticancer effects under continuous high-dose exposure, while clinical applications remain limited to palliative endpoints due to pharmacokinetic variability, bioavailability issues, and regulatory barriers. The central bridge highlights translational challenges, and the bottom strip emphasizes future directions such as nanotechnology-based delivery, standardized dosing, and randomized controlled trials.

Despite compelling preclinical evidence supporting the anticancer potential of *Cannabis sativa* phytochemicals, clinical translation remains limited. Figure 13 illustrates this disconnect, contrasting the robust effects observed in cell and animal models such as apoptosis induction, oxidative stress modulation, and angiogenesis inhibition with the palliative endpoints emphasized in clinical trials. The schematic highlights key translational challenges, including pharmacokinetic/pharmacodynamic mismatch, safety concerns, lack of randomized controlled trials, and strain variability. These barriers underscore the need for innovative drug delivery systems and standardized clinical protocols to bridge the gap between experimental efficacy and therapeutic viability. As research progresses, incorporating nanotechnology-based formulations, targeted delivery methods, and comprehensive clinical designs will be crucial to fully realize the potential of cannabis-derived compounds in oncology.

## 10 Controversies and conflicting evidence in cannabis cancer research

Despite promising preclinical evidence, cannabis-derived compounds present a complex and sometimes contradictory picture in oncology. Variability in receptor expression is one of the most debated issues. CB_2_ receptor upregulation has been linked to poor prognosis in HER2-positive breast cancer and other malignancies (Ramer et al., 2021), yet other studies associate higher CB_2_ levels with improved survival in lung cancer, hepatocellular carcinoma, and mobile tongue squamous cell carcinoma (Preet et al., 2011; Song et al., 2023). Similarly, CB_1_ receptor expression shows inconsistent patterns across tumour types, with reports of decreased levels in glioma (Pagano et al., 2021), progressive increases in ovarian tumours (Messalli et al., 2014), and variable expression depending on the dualistic model of ovarian carcinogenesis (Ronchi et al., 2021). These discrepancies underscore the tumour-dependent nature of cannabinoid receptor signaling and the need for context-specific interpretation.

Another controversy lies in the so-called “THC paradox.” Δ^9^-tetrahydrocannabinol (THC) has been shown to induce apoptosis via ceramide accumulation and suppression of survival pathways in glioma cells (Gomez del Pulgar et al., 2002), yet in other contexts it promotes angiogenesis and immunosuppression, raising concerns about dose- and environment-dependent effects (Chayasirisobhon, 2021). This duality complicates its therapeutic positioning in oncology.

Debate also persists over the relative efficacy of full-spectrum extracts compared to isolated cannabinoids. Whole-plant preparations may benefit from synergistic “entourage effects” (AlmogiHazan and Or, 2020), but reproducibility is poor due to strain variability and inconsistent phytochemical profiles (Koltai and Shalev, 2022). These inconsistencies hinder regulatory approval and clinical translation.

Clinical evidence adds further uncertainty. Human trials remain largely focused on palliative endpoints such as pain relief, appetite stimulation, and nausea control (Aly et al., 2019), with limited data on direct anticancer efficacy. Bioavailability issues and inconsistent absorption further complicate therapeutic application (Kopustinskiene et al., 2020; Johnson et al., 2020). Methodological challenges compound these problems, as differences in cell lines, animal models, and delivery methods hinder cross-study comparisons (Cherkasova et al., 2022; Laezza et al., 2020), while regulatory restrictions continue to limit large-scale, standardized investigations (Capodice and Kaplan, 2021).

Taken together, these controversies highlight the complexity of cannabis-based oncology research. They reinforce the urgent need for standardized formulations, mechanistic clarity, and rigorous clinical designs to resolve contradictions and establish the true therapeutic potential of cannabis-derived compounds in cancer treatment. These controversies highlight the complexity of cannabis-based oncology research and reinforce the need for innovative strategies to resolve contradictions. Building on this critical evaluation, the following section explores prospects, focusing on nanotechnology-enhanced delivery systems, standardized dosing protocols, and rigorous clinical trials that may transform cannabis-derived compounds from experimental promise into viable oncological therapies.

## 11 Prospects

*Cannabis sativa* is emerging as a promising adjunct therapy in oncology due to its diverse bioactive compounds, including cannabinoids, flavonoids, and terpenes, which exhibit anticancer properties through apoptosis induction, tumour suppression, and immune modulation. While preclinical studies highlight its therapeutic potential, clinical integration faces challenges related to standardization, bioavailability, and regulatory constraints.

Future research should focus on further mechanistic insights, particularly investigating cannabinoid interactions with key cancer pathways such as PI3K/AKT/mTOR, MAPK/ERK, and Wnt/β-catenin, to refine precision oncology applications. Comparative analyses between synthetic cannabinoids and full-spectrum extracts could determine optimal efficacy and guide therapeutic strategies.

Additionally, advancements in drug delivery via nanotechnology and liposomal formulations may enhance cannabinoid stability and tumour specificity, while transdermal and inhalable delivery systems offer improved absorption and minimized systemic side effects.

Overcoming resistance mechanisms remains critical, with studies exploring synergies between cannabinoids and chemotherapy, immunotherapy, and radiotherapy to counteract cancer cell adaptation. Combining cannabis-derived terpenes with flavonoids could further enhance cytotoxicity, expanding treatment options.

Moreover, large-scale randomized controlled trials (RCTs) are necessary to validate efficacy and safety, alongside standardized dosing guidelines to support oncologists integrating cannabinoid therapies. While C. sativa presents exciting possibilities for improving cancer treatment outcomes, scientific rigor, regulatory progress, and innovative drug development are essential to unlock its full potential. Continued research will determine its clinical viability, ensuring it evolves from experimental studies to mainstream oncology care.

## 12 Conclusion

This review underscores the diverse and context-dependent role of *Cannabis sativa* phytochemicals in oncology. By first presenting the major oncogenic pathways and then mapping how cannabinoids and flavonoids modulate these signaling cascades, we provide a structured framework that highlights both mechanistic insights and therapeutic promise. Cannabinoids such as Δ9-tetrahydrocannabinol (THC), cannabidiol (CBD), and cannabigerol (CBG) exert pathway-specific effects on apoptosis, oxidative stress regulation, autophagy, angiogenesis, and metastasis (Massi et al., 2013; Shrivastava et al., 2011; Borrelli et al., 2014; Moreno et al., 2014; Tomko et al., 2020). Flavonoids, including cannflavin A, genistein, daidzein, hesperetin, and naringenin, demonstrate selective cytotoxicity across bladder, breast, melanoma, and pancreatic cancers, often sparing normal tissue (Tomko et al., 2022; Kaushik et al., 2019; Kopustinskiene et al., 2020; Jin et al., 2010; Sohel et al., 2022; Choi et al., 2020; Moreau et al., 2019).

Importantly, the interactions among phytochemicals are not uniformly synergistic. While combinations such as THC and CBD amplify apoptotic signaling beyond individual effects (Torres et al., 2011), others act additively or even antagonistically (Russo, 2011; Borrelli et al., 2014). Recognizing these distinctions strengthens the scientific basis for multi-component *cannabis*-based therapies and cautions against oversimplifying the “entourage effect.” Clinical formulations such as Nabiximols provide translational evidence of cannabinoid synergy, though outcomes remain context-dependent (Johnson et al., 2010).

Future research should prioritize mechanistic and translational studies, paying attention to dose optimization, rational phytochemical combinations, and innovative drug delivery systems (Moreau et al., 2019). It is also essential to clarify terminology: ADME profiling represents a set of *in vitro* protocols designed to predict pharmacokinetic and pharmacodynamic properties, but it does not equate to drugability (Laezza et al., 2020). Rigorous preclinical and clinical evaluation remains indispensable to determine whether *Cannabis sativa* phytochemicals can be advanced into safe and effective anticancer therapies.

Taken together, these findings position *Cannabis sativa* phytochemicals not merely as natural products of interest, but as promising molecular entities with the potential to reshape integrative oncology.
